# Clinical and 18F-FDG PET/CT Imaging Characteristics of Post-radiotherapy Sacral Insufficiency Fractures in Cervical Cancer Patients

**DOI:** 10.2174/0115734056428627260223054010

**Published:** 2026-04-08

**Authors:** Wanling Qi, Fengxiang Liao, Zhehuang Luo, Mingyan Shao

**Affiliations:** 1 Department of Nuclear Medicine, The First Affiliated Hospital of Nanchang Medical College, Jiangxi Provincial People’s Hospital, Nanchang, China

**Keywords:** Sacrum, Insufficiency fracture, Fluorodeoxyglucose, Positron emission computed tomography, Cervical cancer, Radiotherapy

## Abstract

**Introduction:**

Sacral Insufficiency Fractures (SIFs) are a common yet frequently misdiagnosed late complication following pelvic radiotherapy for cervical cancer. Accurate differentiation from bone metastases is crucial to avoid unnecessary interventions. While MRI is sensitive for early marrow edema, integrated 18F-FDG PET/CT offers a unique simultaneous assessment of bone metabolism and systemic tumor status. However, comprehensive studies detailing the qualitative and quantitative PET/CT characteristics of post-radiotherapy SIFs are lacking. This study aims to systematically define these features and establish their discriminative value.

**Materials and Methods:**

In this retrospective study, we analyzed 32 cervical cancer patients who developed SIFs following pelvic radiotherapy and underwent 18F-FDG PET/CT imaging between January 2018 and January 2024. Diagnosis was based on characteristic radiologic findings, clinical correlation, and a minimum 12-month follow-up. Qualitative (fracture patterns, FDG uptake morphology) and quantitative (SUVmax, SUR-BP ratio, CT densitometry) parameters on 18F-FDG PET/CT were evaluated.

**Results:**

SIFs predominantly occurred in postmenopausal women (93.7%, 30/32) at a median of 14 months post-radiotherapy. Sacral involvement was observed as follows: unilateral ala (53.1%, 17/32), bilateral alae (37.5%, 12/32), and extension to the sacral body (9.4%, 3/32). The affected segments were primarily located at S1-S3. Concomitant pelvic fractures were also frequently identified, including pubic (28.1%, 9/32), iliac (25%, 8/32), and bilateral L5 transverse process fractures (6.2%, 2/32). Common CT findings included ill-defined osteosclerosis near the sacroiliac joint (87.5%, 28/32) and linear or curvilinear hypoattenuating fracture lines (68.7%, 22/32). PET revealed characteristic mild, diffuse/patchy FDG uptake parallel to the sacroiliac joint (96.8%, 31/32) with low metabolic activity (mean SUVmax 2.45±0.74, mean SUR-BP 1.46±0.38). Quantitative CT confirmed significant osteopenia within the radiation field (mean HU 36.8±28.6 *vs.* 78.0±37.3 outside, *p*<0.001).

**Discussion:**

Post-radiation SIFs predominantly affect postmenopausal cervical cancer patients due to radiotherapy-induced osteoporosis and bone vulnerability. These fractures often present with nonspecific pain and require differentiation from bone metastases, for which 18F-FDG PET/CT is essential due to its ability to detect characteristic metabolic patterns and associated osteoporotic changes. Key diagnostic features include linear or curvilinear hypoattenuating fracture lines, mild diffuse/patchy FDG uptake parallel to the sacroiliac joint, and background osteoporotic changes within radiation fields. Integrated PET/CT outperformed single-modality imaging by enabling simultaneous assessment of bone metabolism and systemic tumor status, a critical advantage over MRI (superior for marrow edema but unable to evaluate systemic disease) and standalone CT (lacking metabolic discrimination of benign vs malignant lesions). Early recognition of SIFs through integrated imaging is critical to avoid misdiagnosis and unnecessary invasive procedures, thereby guiding appropriate conservative management.

**Conclusion:**

SIFs represent a prevalent post-radiotherapy complication in cervical cancer patients, with a particular predilection for postmenopausal women. 18F-FDG PET/CT demonstrates high diagnostic reliability for diagnosing SIFs, which typically present as linear fractures parallel to the sacroiliac joints on a background of osteoporotic changes, accompanied by mild diffuse or patchy FDG uptake and frequently co-occurring with pelvic fractures at other sites. Integrated PET/CT is crucial for early recognition, preventing misdiagnosis as metastasis, and guiding appropriate conservative management.

## 1 INTRODUCTION

Insufficiency Fractures (IFs) are a type of stress fracture caused by normal or physiological stress, which occurs when such stress acts on bones with demineralization or impaired elasticity. The sacrum, a key component of the posterior pelvic ring, is the most common site of IFs, accounting for 75% of all such lesions, owing to its critical weight-bearing role and inherent tendency for stress concentration [1-3]. Sacral Insufficiency Fractures (SIFs) were first described in 1982 by Lourie *et al*. in osteoporotic elderly patients presenting with spontaneous sacral fractures and associated back and leg pain [2, 4]. SIFs are now known to predominantly affect postmenopausal women with osteoporosis. Additional risk factors include rheumatoid arthritis, chronic glucocorticoid use, diabetes mellitus, cancer therapies (radiotherapy, chemotherapy), and emerging associations with low BMI (Body Mass Index), multiparity, and smoking [5, 6]. Notably, pelvic radiotherapy represents a major iatrogenic contributor, demonstrating a significantly higher SIFs incidence (8.2 to 45.2%) in irradiated *versus* non-irradiated patients, with risk influenced by radiation dose and technique [7-9]. In cervical cancer patients, pelvic radiotherapy is a standard curative or adjuvant treatment, and its widespread use over the past 15-20 years has been accompanied by a rising incidence of post-radiotherapy SIFs. The non-specific symptoms (often low back/pelvic pain) and imaging overlap with bone metastases create a high risk of misdiagnosis, potentially leading to unnecessary invasive procedures or toxic therapies. Conversely, delayed diagnosis may lead to progressive pain, vertebral instability, and even neurological deficits from sacral nerve compression. Therefore, early and accurate identification of post-radiotherapy SIFs is crucial for optimizing patient outcomes and preventing avoidable harm.

The primary pathological manifestations of SIFs include repeated microfractures and repair of cancellous bone, leading to glucose consumption by osteoblasts and inflammatory cells. These glucose metabolic changes can be sensitively detected by fluorodeoxyglucose (18F-FDG) Positron Emission Tomography (PET) imaging. Concurrent low-dose Computed Tomography (CT) provides complementary structural assessment of bone density and morphology, establishing PET/CT as an effective SIFs diagnostic tool [10-13]. While prior studies [14-16] have characterized SIFs' appearances on 99Tcm-MDP bone scintigraphy, Computed Tomography (CT), and Magnetic Resonance Imaging (MRI), data on 18F-FDG PET/CT manifestations remain limited. Currently available studies lack systematic quantitative metabolic metrics and fail to correlate imaging features with radiation-induced osteopenia. Moreover, the distinct value of integrated PET/CT compared to single-modality imaging (such as MRI or CT) has not been clearly established in the setting of post-radiotherapy follow-up for cervical cancer.

The present study aims to systematically characterize the clinical and 18F-FDG PET/CT imaging characteristics (both qualitative morphological patterns and quantitative metabolic parameters) of post-radiotherapy SIFs in cervical cancer patients. Specifically, we seek to (1) provide comprehensive quantitative metabolic (SUVmax, SUR-BP) and structural (CT densitometry) benchmarks, (2) directly correlate imaging findings with objective evidence of radiation-induced osteopenia, (3) explicitly define the discriminative PET/CT features to differentiate SIFs from bone metastases, and highlight the integrated modality's unique clinical utility over MRI or CT alone. By addressing these objectives, we aim to enhance clinical recognition of this common radiotherapy complication and reduce the burden of misdiagnosis in cervical cancer follow-up.

## 2 MATERIALS AND METHODS

### 2.1 Ethical Approval

This single-center, retrospective study analyzed clinical data and 18F-FDG PET/CT parameters obtained from electronic medical records. The study was determined to be exempt from formal ethical review by the Institutional Ethics Committee of Jiangxi Provincial People’s Hospital, as it involved only the analysis of pre-existing, anonymized data and posed minimal risk to participants, in accordance with applicable guidelines and the ethical principles of the 1964 Declaration of Helsinki and its later amendments. Due to the retrospective design and use of anonymized data, the requirement for informed consent was also waived.

### 2.2 Study Subjects

Cervical cancer patients post-radiotherapy who underwent 18F-FDG PET/CT at Jiangxi Provincial People’s Hospital between January 2018 and January 2024 were screened. From 41 initially enrolled, 9 were excluded (5 for poor image quality, 4 for follow-up <12 months). The final cohort comprised 32 female patients (age: 47-83 years, mean 63.88±9.38). Inclusion criteria included (1) pathology-proven cervical cancer, and received radiotherapy (RT), (2) baseline 18F-FDG PET/CT or CT data from before radiotherapy, and (3) compared with baseline CT, new fracture lines and/or sclerosis of the sacrum, and (4) increased metabolic activity in the sacral region, with no evidence of bone metastases (such as osteolytic bone destruction or soft tissue masses) during a minimum 12-month follow-up period. Exclusion criteria included (1) having sacrum metastasis, and (2) having a history of pelvic trauma.

### 2.3 Scan Technique

A standardized protocol was used, with patients fasting for≥6 hours (blood glucose <7.4 mmol/L), and after intravenous injection of 5.6 MBq/kg 18F-FDG, a 45-60 minute uptake period ensued. Imaging was performed on a GE Discovery STE PET/CT scanner. A low-dose CT (120 kV, auto mA, pitch 1.75:1, 16×3.75 mm collimation, 0.5 s rotation) from skull vertex to upper thighs was acquired first for attenuation correction and anatomy. No contrast was used. PET data were then acquired in 3D mode (2.5 min/bed position, 6-7 beds). Images were reconstructed using CT-based attenuation correction and the OSEM algorithm (2 iterations, 28 subsets) with an 8-mm Gaussian filter onto a 128×128 matrix. Analysis used a GE AW 4.6 PACS workstation for multiplanar PET, CT, fused PET/CT, and MIP views.

### 2.4 Data Analysis

Two blinded attending physicians (15 and 10 years of PET/CT experience) independently evaluated images. Disagreements were resolved by consensus. The analysis included the following:

(1) Observation and documentation of the sacral fracture pattern, involvement scope, presence of fracture lines, CT bone changes, *etc.*, and document whether there are concomitant fractures in the thoracolumbar spine and other parts of the pelvis; (2) Assessment of the FDG uptake pattern in the lesion area and the maximum standard uptake value (SUVmax); liver blood pool was selected as the background, The mean Standardized Uptake Value of the liver (SUVmean) was used as the blood pool SUVmean, and the ratio of the lesion SUVmax to the liver blood pool SUVmean (Standardized Uptake Ratio–Blood Pool, SUR-BP) was calculated; (3) CT values of lumbar vertebrae in the radiation area and CT values of vertebrae outside the adjacent radiation area were measured; measurement method: vertebrae with uniform density in the corresponding area was selected, and the largest possible region of interest (ROI) was set, bone islands, sclerosing areas, bone cortex, and regions with trabecular deficiency at the posterior vertebral venous entrance were avoided. The average value was obtained after independent measurements by two physicians.

### 2.5 Statistical Analysis

All data are presented as mean ± standard deviation. Descriptive statistics, including frequencies, means, and medians, were used to analyze patients’ clinical data and scan results. All statistical analyses were conducted using SPSS version 26.0 for Windows.

## 3 RESULTS

### 3.1 Clinical Features

In this study, all 32 patients were female, with ages ranging from 47 to 83 years (mean age 63.88 ± 9.38 years; median age 66 years). SIFs occurred predominantly in postmenopausal patients, accounting for 93.7% (30/32) of patients. The median time from radiotherapy initiation to imaging detection of SIFs was 14 months (range, 2-32 months), and the great majority of affected patients developed SIFs within 2 years after RT (90.6%, 29 of 32 patients). Among the patients, 18 (56.3%) presented with low back or hip pain, and 5 (15.6%) were asymptomatic and detected during routine oncological surveillance. In comparison, the remaining 9 (28.1%) were identified on imaging due to tumor recurrence (including irregular vaginal bleeding in 3 cases, abdominal pain in 3, low back pain in 2, and fecal discharge from the vagina in 1). The demographic data and clinical characteristics associated with SIFs were summarized in Table **1**. All 32 cervical cancer patients had no history of trauma or malignant tumors in other systemic sites. Of these, 22 patients underwent hysterectomy with bilateral salpingo-oophorectomy followed by radiotherapy, while 10 patients received radical radiotherapy alone.

### 3.2 PET/CT Findings

**Fig. (1):**
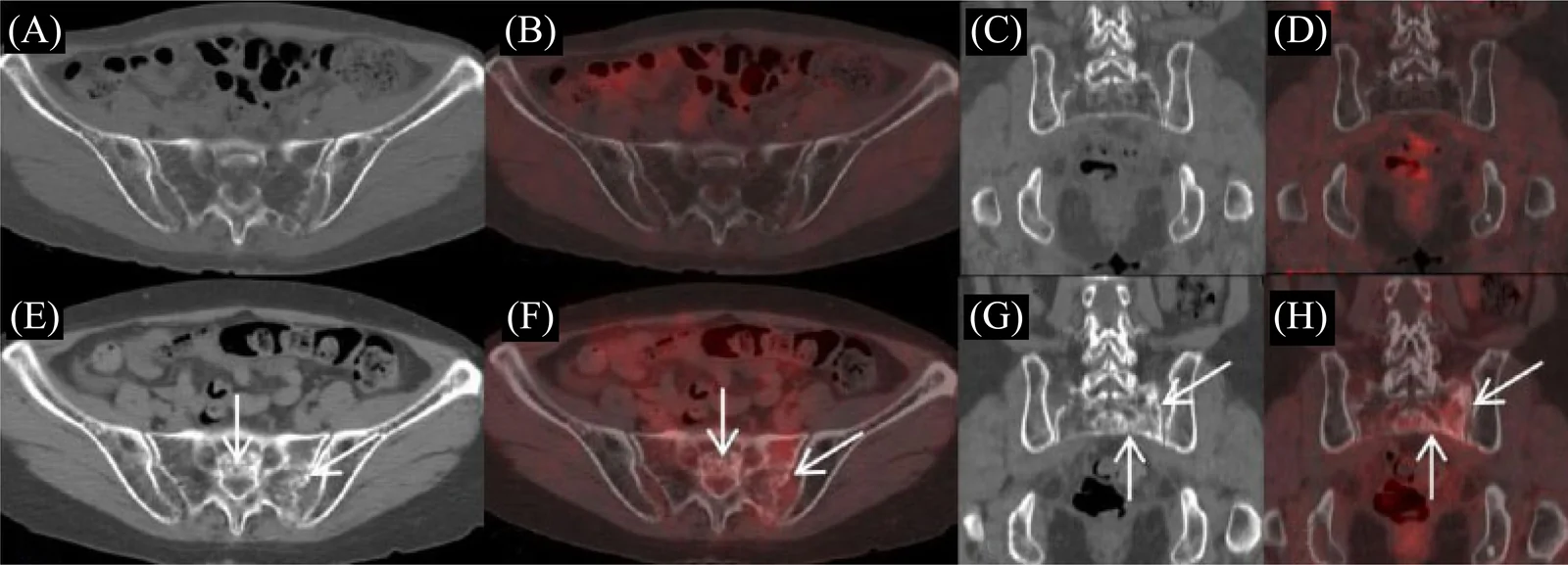
A 68-year-old female with SIFs at 30 months after radiotherapy for cervical cancer, presenting with hip pain for more than 1 month. At 10 months post-radiotherapy, axial and coronal CT images (**A**, **C**) showed decreased bone mineral density of the pelvis, presenting as osteoporotic changes, and the corresponding axial and coronal PET/CT fusion images (**B**, **D**) revealed no abnormal increased FDG uptake. Axial and coronal CT images (**E**, **G**) at 30 months post-radiotherapy demonstrated irregular low-density fracture lines in the left sacral ala and sacral body, accompanied by multiple ill-defined patchy slightly hyperdense/high-density shadows in the surrounding area (arrow, ↑), while the corresponding axial and coronal PET/CT fusion images (**F**, **H**) showed diffusely mild increased FDG uptake with a SUVmax of 3.0 (arrow, ↑).

**Fig. (2):**
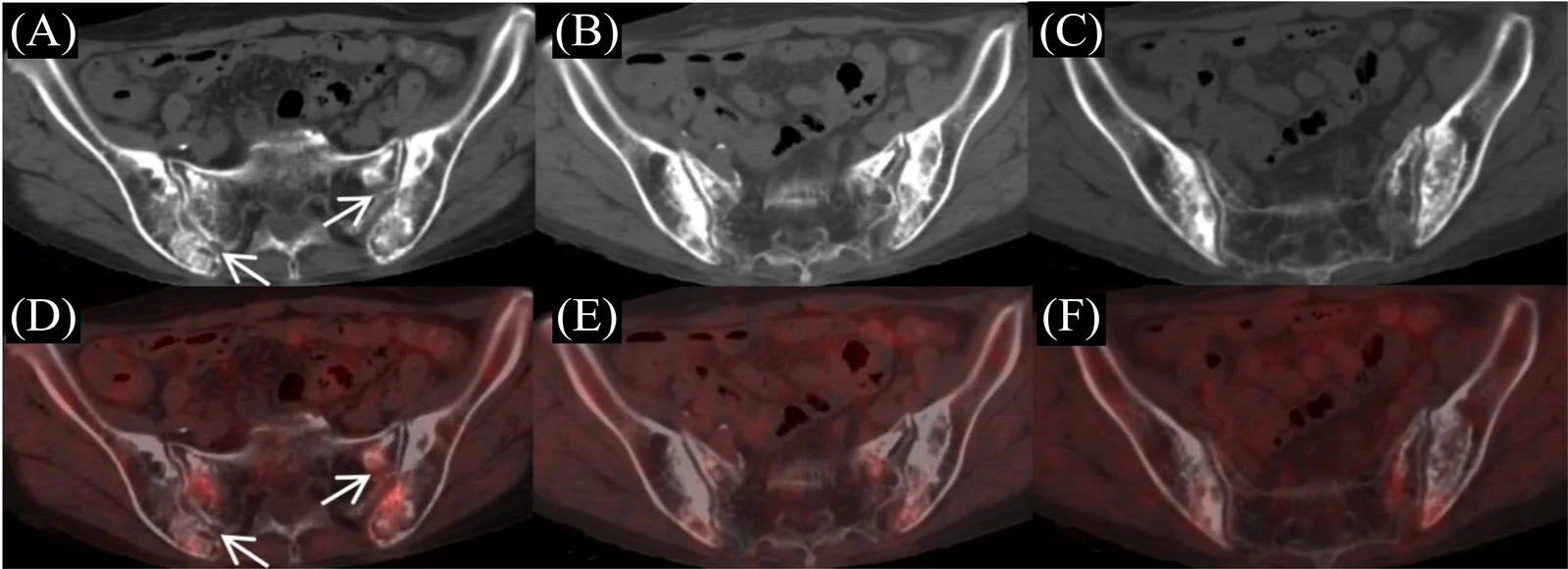
A 67-year-old female with SIFs at 16 months after radiotherapy for cervical cancer, presenting with vaginal fecal fluid leakage for more than 1 month. Axial CT images (**A**-**C**) showed fracture lines or cortical buckling in the bilateral sacral alae and bilateral ilia, with multiple nodular and patchy hyperdense shadows in the surrounding area, presenting as obvious proliferative sclerosis with relatively clear boundaries (arrow ↑). The corresponding axial PET/CT fusion images (**D**-**F**) revealed multiple scattered patchy areas of mildly increased FDG uptake, with a SUVmax of 2.0.

Among the 32 patients, sacral involvement patterns were as follows: unilateral left sacral ala in 9 cases (28.1%), unilateral right sacral ala in 8 cases (25%), bilateral sacral alae in 12 cases (37.5%), left sacral ala with sacral body involvement in 1 case (3.1%) (Fig. **1**), and bilateral sacral alae with sacral body involvement in 2 cases (6.2%). The concomitant fractures were observed in 9 patients (28.1%) with pubic fractures, 8 cases (25%) with iliac fractures (Fig. **2**), and 2 patients (6.2%) with bilateral L5 transverse process fractures. The affected sacral vertebrae ranged from S1 to S3, and no associated soft-tissue masses were identified.

**Fig. (3):**
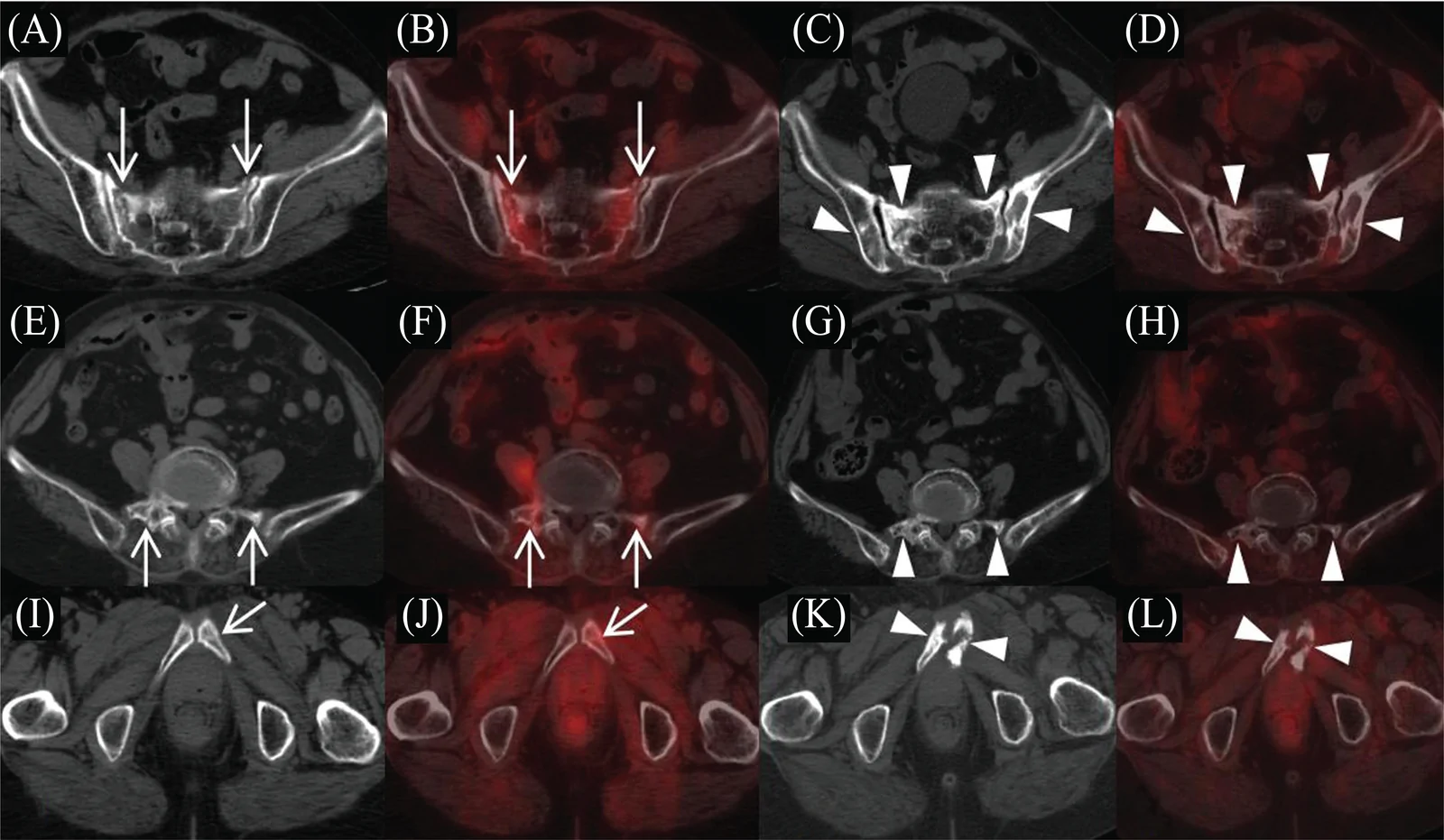
A 72-year-old female with SIFs at 9 months after radiotherapy for cervical cancer, presenting with low back pain for more than 2 months. Axial CT images (**A**, **E**, **I**) showed low-density fracture lines or cortical buckling in the bilateral sacral alae, bilateral transverse processes of L5, left pubic ramus, and pubic symphysis; among these, the fracture lines in the sacral alae were serrated and parallel to the sacroiliac joints (arrow ↑). Multiple ill-defined, patchy, slightly hyperdense shadows were observed around the fracture lines. The corresponding axial PET/CT fusion images (**B**, **F**, **J**) demonstrated diffusely mild increased FDG uptake with a SUVmax of 3.4 (arrow ↑). At the follow-up examination at 32 months after radiotherapy, axial CT images (**C**, **G**, **K**) revealed that the range of abnormal bone changes had significantly enlarged compared with the previous findings, showing multiple punctate and patchy hyperdense shadows with obvious proliferative sclerosis and clear boundaries in the bilateral regions.Sacral alae, bilateral ilia, bilateral transverse processes of L5, bilateral pubic rami, and pubic symphysis; only the fracture ends of the left pubic ramus and pubic symphysis were obviously separated, while the fracture lines in other locations had disappeared, showing post-repair changes (arrow ▲). The corresponding axial PET/CT fusion images (**D**, **H**, **L**) showed a decreased SUVmax of 1.4 (arrow ▲).

**Fig. (4):**
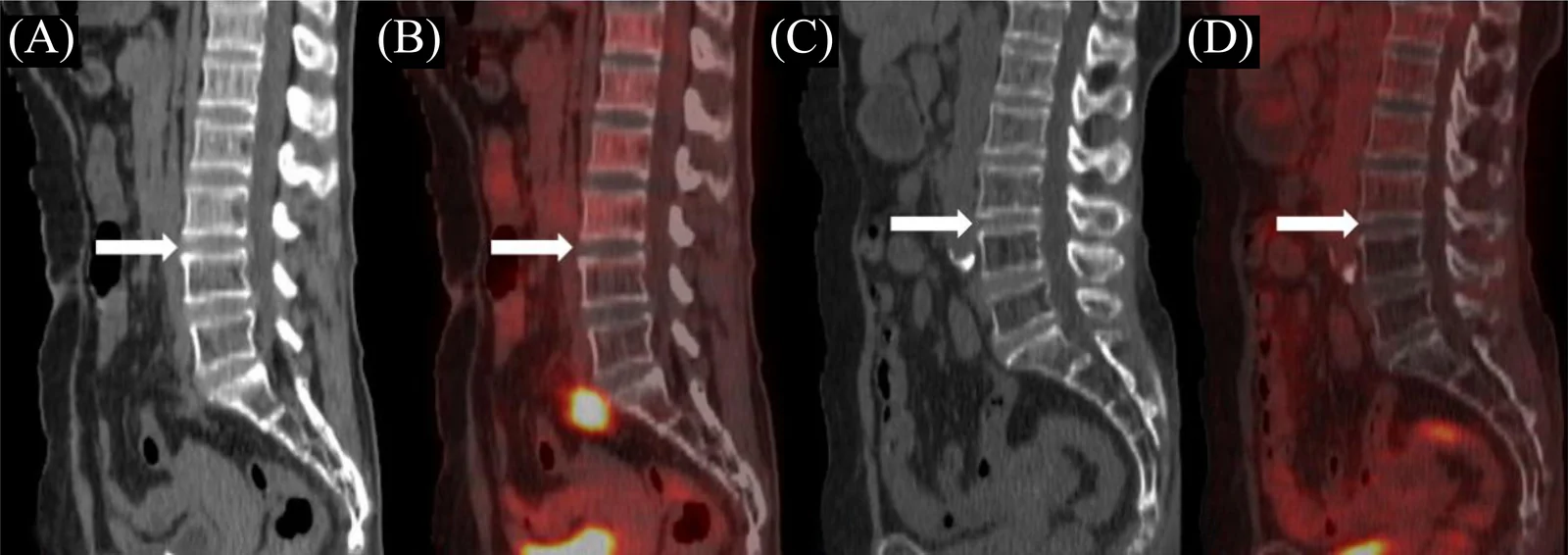
Sagittal CT and PET/CT fusion images demonstrate radiation-induced Sacral Insufficiency Fractures (SIFs) in two cervical cancer patients post-radiotherapy. In the 52-year-old female (7 months post-RT), CT reveals markedly reduced vertebral density within the irradiated field (39 HU) compared to non-irradiated bone (110 HU) at the L3-L4 boundary (arrow ↑, **A**), with correspondingly lower FDG uptake (SUVmax 0.56 *vs.* 1.65; arrow, **B**). Similarly, the 65-year-old female (13 months post-RT) shows more pronounced osteopenia in irradiated vertebrae (5 HU *vs.* 59 HU; arrow, **C**) and suppressed metabolic activity (SUVmax 0.70 *vs.* 2.01; arrow, **D**), both delineated by the L3-L4 interface.

Regarding imaging characteristics, 28 patients (87.5%) exhibited heterogeneous bone density elevation adjacent to the sacroiliac joint, characterized by ill-defined margins around the hyperdense regions; the remaining 4 patients showed normal bone density. Linear or curvilinear hypoattenuating fracture lines were observed in 22 patients (68.7%). Hypermetabolism was observed in all patients, with the SUVmax ranging from 1.5 to 5.1, an average of 2.45 ± 0.74, and a median of 2.4. In one case, a follow-up PET/CT scan performed two years later demonstrated progressive osteosclerosis with increased bone density and an expanded lesion area. Notably, the SUVmax declined markedly from 3.4 to 1.4, suggesting reduced metabolic activity (Fig. **3**). A diffuse or patchy uptake pattern parallel to the sacroiliac joint was seen in 31 patients (96.8%), which did not fully coincide with the areas of increased bone density; the remaining patient presented with focal metabolic hyperactivity. The standardized uptake ratio relative to the liver blood pool (SUR-BP) exhibited a range of 1.3 to 2.0, with a mean value of 1.46 ± 0.38 and a median value of 1.47. CT analysis revealed significant differences in vertebral body attenuation values between the regions within and outside the SIFs radiotherapy field, with mean CT values of 36.8±28.6 HU and 78.0±37.3 HU, respectively (paired t-test: t=-4.950, *P*<0.001) (Fig. **4**). The PET/CT imaging characteristics associated with SIFs were summarized in Table **2**.

## 4 DISCUSSION

Cervical cancer ranks as the fourth most common female malignancy globally, and radiotherapy is commonly employed in its clinical management, either as a standalone therapeutic modality or as adjuvant irradiation following surgical resection [17, 18]. Post-radiation SIFs have emerged as a common sequela, yet their clinical recognition remains suboptimal. Our study confirmed that SIFs predominantly affect postmenopausal women with a history of pelvic radiotherapy, consistent with the conclusions of earlier published reports. Firstly, age and menopausal status are key risk factors: prior evidence indicates that postmenopausal women over 55 are most susceptible to SIFs, a pattern reflected in our cohort, where 93.7% of SIF patients were postmenopausal (median age: 66.2 years). This is because postmenopausal estrogen deficiency exacerbates radiation-induced bone loss [19], and age-related muscle atrophy further compromises the protective effect of muscles on bone [20, 21]. Secondly, radiotherapy-related bone damage: pelvic radiotherapy exacerbates bone vulnerability by directly damaging osteoblasts, osteocytes, and microvasculature, inducing vascular occlusion, bone necrosis, and secondary osteoporosis [22]. Our quantitative CT densitometry data (irradiated vertebrae: 36.8±28.6 HU *vs.* non-irradiated: 78.0±37.3 HU, *p*<0.001) provide objective validation of radiotherapy-induced osteopenia. Thirdly, cumulative treatment burden: Although Gondi *et al*. [23] documented that CCRT (*versus* sole radiotherapy) increases susceptibility to SIFs due to cisplatin-mediated bone marrow suppression and impaired osteoclast-osteoblast balance, the exact causal relationship and underlying regulatory mechanisms linking CCRT to SIFs occurrence remain poorly defined in the current literature.

Clinically, SIFs present with nonspecific lumbopelvic or lower extremity pain, which is often exacerbated by activity. In oncology patients, this symptom profile frequently raises concern for disease recurrence. Indeed, 62.5% of our patients reported persistent discomfort, underscoring the need to consider SIFs in symptomatic, post-radiotherapy patients. The reported incidence of post-radiotherapy SIFs varies widely (8.2-45.2%), depending on radiation dose, technique, and follow-up duration [7-9]. Radiation-induced osteopenia and subsequent fracture risk are also influenced by the radiotherapy technique used. For example, Sapienza *et al*. [7] demonstrated a lower incidence of pelvic insufficiency fractures with intensity-modulated radiotherapy (IMRT) compared to 3D conformal radiotherapy (3D-CRT), likely due to the larger pelvic bone volume exposed to high-dose radiation in 3D-CRT. When compared with other cancer populations, the 5-year incidence of pelvic fractures differs. It is lower in gynecologic cancer patients (11.8%) than in gastrointestinal cancer patients (12.7%), but higher than in prostate cancer patients (3.7%) [24]. The time to onset of SIFs is similarly variable. In our cohort, the median time to diagnosis was 14 months (range 2-32 months), consistent with the 6-20 month median reported in other series [25]. This timing may be influenced by baseline bone health and adjuvant therapies. For instance, patients with significant pre-existing osteoporosis or those receiving higher radiation doses may present earlier. The detection of fractures as early as 2 months post-radiotherapy supports the concept that acute vascular injury can precipitate microfractures in osteoporotic bone [26]. Our maximum follow-up of 32 months may not have captured very late-onset cases, underscoring the need for prolonged surveillance in high-risk populations. Management of SIFs is generally conservative, although surgical intervention may be required for unstable fractures [27].

Imaging plays a central role in diagnosis. (1) MRI remains superior for detecting the earliest marrow edema of occult SIFs [15]. However, as demonstrated here, integrated PET/CT offers the unique and critical advantage of simultaneous whole-body tumor restaging. In an oncology patient with new back pain, a single PET/CT scan can both diagnose SIFs and rule out metastatic disease, a decisive capability MRI lacks. (2) While CT shows fracture morphology and osteopenia, it lacks metabolic information. Our data show that 12.5% of SIFs had no definitive CT abnormality initially, yet were PET-positive. PET/CT's metabolic data is crucial for identifying these early/active fractures and for differentiating benign sclerotic changes from malignancy. (3) Though sensitive, bone scintigraphy lacks the specificity and anatomical detail provided by CT fusion in PET/CT.

Our PET/CT findings both align with and extend previous reports on post-radiotherapy SIFs. The most consistent imaging feature was a linear or curvilinear fracture line parallel to the sacroiliac joint within the sacral ala [10-14, 28]. When concurrent involvement of the sacral body is present, this pattern corresponds to the classic “Honda sign” or its variants as described in bone scintigraphy [29]. A notable observation was that 46.9% of patients had concomitant pelvic fractures (pubic, iliac, or L5), emphasizing that SIFs are often part of a broader pelvic insufficiency injury pattern, as noted in other studies [10-14, 30]. Regarding metabolic activity, our quantitative data (mean SUVmax 2.45±0.74) align with several case series reporting mild to moderate FDG uptake (SUVmax 1.1-2.9) [31, 32] but are lower than the higher ranges (5.2-8.3) reported by Ravenel *et al*. [30]. In this study, one patient showed a significant reduction in metabolism on follow-up PET/CT examination two years later. This variability likely reflects differences in the timing of imaging relative to the fracture's healing phase, a relationship that remains to be fully characterized. We provide new data on the SUR-BP ratio (mean 1.46±0.38), offering a potentially more stable metric for gauging metabolic activity relative to background. Importantly, we observed that metabolic changes on PET could precede definitive sclerotic changes on CT in some cases, a finding that underscores PET's sensitivity to the early reparative inflammatory process. However, it does not surpass MRI for detecting the very earliest marrow edema.

Accurate differentiation between SIFs and bone metastases in post-radiotherapy cervical cancer patients is critical. Misdiagnosis of SIFs as metastasis can lead to unnecessary biopsies or aggressive systemic therapy, while missing a true metastasis delays appropriate treatment. Integrated PET/CT offers key discriminators, synthesizing findings from both modalities. (1) Metabolic Pattern and Intensity: SIFs typically show mild, linear, or diffuse FDG uptake along fracture lines. In contrast, bone metastases usually demonstrate focal, nodular, or mass-like intense uptake. An SUVmax threshold of 5.0 has shown good diagnostic performance for this differentiation [33, 34], and our cohort's values fell well below this. (2) Morphological Background on CT: SIFs occur within a context of diffuse radiation-induced osteopenia and often show ill-defined reparative sclerosis. Metastases, whether osteolytic or osteoblastic, typically present as more focal, destructive, or blastic lesions without generalized osteopenia in the surrounding bone. (3) Ancillary Findings: The presence of other pelvic insufficiency fractures and the classic fracture morphology are strongly suggestive of SIFs.

None of the patients in this study underwent biopsy, as this procedure is not recommended owing to the high possibility of fracture and low diagnostic value. Moreover, radiation-induced bone marrow hypovascularization increases the risk of biopsy-site infection, which may subsequently lead to osteonecrosis [35]. Diagnosis can typically be confirmed based on clinical and radiological findings, as clinical symptoms typically improve over time in conjunction with radiographic regression. However, it is noteworthy that new fractures may arise in non-irradiated areas as well.

In summary, 18F-FDG PET/CT is a highly valuable tool in the post-radiotherapy setting. Its integrated nature allows for the reliable diagnosis of SIFs based on a combination of characteristic metabolic patterns and CT morphology within an osteoporotic background, while simultaneously performing essential whole-body restaging to exclude metastatic disease. This one-step comprehensive assessment is a significant advantage over sequential or single-modality imaging.

## 5 STUDY LIMITATIONS

While this study provides valuable insights into 18F-FDG PET/CT characteristics of post-radiotherapy SIFs, several limitations should be noted. The retrospective design may introduce selection bias, the limited sample size limits statistical power, and the inherent overlap in FDG avidity between benign fractures and malignant lesions challenges diagnostic specificity. Additionally, variations in imaging protocols and a lack of standardized follow-up may affect reproducibility. Future prospective multicenter studies incorporating advanced imaging modalities (*e.g.*, PET/MRI) and quantitative biomarkers are needed to enhance diagnostic accuracy and establish clinically relevant SUV thresholds for differentiating fracture-related uptake from tumor recurrence.

## CONCLUSION

SIFs represent a common complication following radiotherapy in cervical cancer patients, with a particular predilection for postmenopausal women. 18F-FDG PET/CT demonstrates high diagnostic reliability for SIFs, typically characterized by linear fractures parallel to the sacroiliac joints on a background of osteoporotic changes, accompanied by mild diffuse or patchy FDG uptake, and frequently co-occurring with pelvic fractures at other sites. Compared to single-modality imaging (*e.g.*, MRI or CT alone), integrated PET/CT is crucial for early recognition of SIFs, preventing misdiagnosis as metastasis, and guiding appropriate conservative management.

## Figures and Tables

**Table 1:** Clinical characteristics of 32 patients with SIFs.

Clinical Data	Statistics
Age	(Years)
Range	47–83
Average age	63.88±9.3
Median age	66
Postmenopausal status, n (%)	30 (93.7%)
Time interval from RT to SIF	(Months)
Range	2–36
Median time	14
Symptoms, n (%)	
Hip/low back pain	18 (56.3%)
Asymptomatic	5 (15.6%)
Tumor recurrence	9 (28.1%)
Irregular vaginal bleeding	3 (9.4%)
Abdominal pain	3 (9.4%)
Low back pain	2 (6.2%)
Fecal discharge from the vagina	1 (3.1%)

**Table 2:** PET/CT imaging findings in 32 patients with SIFs.

Imaging Data	Statistics
Sacral involvement, n (%)	
Unilateral left ala	9 (28.1%)
Unilateral right ala	8 (25.0%)
Bilateral alae	12 (37.5%)
Left ala + body	1 (3.1%)
Bilateral alae + body	2 (6.2%)
Concomitant Fractures, n (%)	
Pubic fractures	9 (28.1%)
Iliac fractures	8 (25%)
Bilateral L5 transverse process fractures	2 (6.2%)
CT density, n (%)	
Increased	28 (87.5%)
Unchanged	4 (12.5%)
Decreased	0 (0%)
Sacral manifestation, n (%)	
Abnormal (fracture lines)	22 (68.75%)
Normal	10 (31.25%)
Metabolism (SUVmax)	
SUVmax (Range)	1.5–5.1
SUVmax (Average)	2.45±0.74
Median SUVmax SUR-BP (Range)	2.41.3-2.0
SUR-BP (Average) Median SUR-BP	1.46±0.381.47
Metabolic pattern, n (%)	
Diffuse or linear	31 (96.8%)
Focal	1 (3.1%)
CT Density Analysis	
Irradiated vertebrae (HU)	36.8 ± 28.6
Non-irradiated vertebrae (HU)	78.0 ± 37.3
*P*-value	<0.001 (t = -4.950)

## Data Availability

The original contributions presented in the study are included in the article. Further inquiries can be directed to the corresponding author [M.S] upon reasonable request.

## LIST OF ABBREVIATIONS

SIFs: Sacral insufficiency fractures
BMI: Body Mass Index
[18F] FDG: 18F-fluorodeoxyglucose
PET: Positron emission tomography
CT: Computed tomography
MRI: Magnetic resonance imaging
RT: Radiotherapy
MIPs: Maximum-intensity projections
SUVmax: Maximum standard uptake value
SUVmean: Mean standard uptake value of the liver
SUR-BP: Standard uptake ratio-blood pool
ROI: Region of interest
CCRT: *Versus* sole radiotherapy
IMRT: Intensity-modulated radiotherapy
3D-CRT: 3D conformal radiotherapy

## STANDARDS OF REPORTING

STROBE guidelines were followed.
